# Patients’ and health system’s delays in the diagnosis and treatment of new pulmonary tuberculosis patients in West Gojjam Zone, Northwest Ethiopia: a cross-sectional study

**DOI:** 10.1186/s12879-016-1995-z

**Published:** 2016-11-11

**Authors:** Senedu Bekele Gebreegziabher, Gunnar Aksel Bjune, Solomon Abebe Yimer

**Affiliations:** 1 Amhara Regional State Health Bureau, Bahir Dar, Ethiopia; 2 Department of Community Medicine, Institute of Health and Society, University of Oslo, Oslo, Norway; 3 Oslo University Hospital, Oslo, Norway; 4 Department of Bacteriology and Immunology, Division of Infectious Disease Control, Norwegian Institute of Public Health, Oslo, Norway

**Keywords:** Tuberculosis, Patients’ delay, Health system’s delay, diagnostic delay, West Gojjam Zone, Ethiopia

## Abstract

**Background:**

Tuberculosis (TB) is a major public health concern in the developing world. Early diagnosis and prompt initiation of treatment is essential for effective TB control. The aim of this study was to determine the length and analyze associated factors of patients’ and health system’s delays in the diagnosis and treatment of new pulmonary TB (PTB) patients.

**Methods:**

A cross-sectional study was conducted in 30 randomly selected public health facilities in West Gojjam Zone, Amhara Region, Ethiopia. Newly diagnosed PTB patients who were ≥15 years of age were consecutively enrolled in the study. Patients’ delay (the time period from onset of TB symptoms to first presentation to a formal health provider) and health system’s delay (the time period from first presentation to a formal health provider to first start of TB treatment) were measured. Median patients’ and health system’s delays were calculated. Mixed effect logistic regression was used to analyze predictors of patients’ and health system’s delays.

**Results:**

Seven hundred six patients were enrolled in the study. The median patients’ delay was 18 days (interquartile range [IQR]: 8–34 days) and the median health system’s delay was 22 days (IQR: 4–88 days). Poor knowledge of TB (adjusted odds ratio [AOR], 2.33; 95 % confidence interval [CI], 1.34–4.05), first visit to non-formal health provider (AOR, 47.56; 95 % CI, 26.31–85.99), self-treatment (AOR, 10.11; 95 % CI, 4.53–22.56) and patients’ age (≥45 years) (AOR, 2.99; 95 % CI, 1.14–7.81) were independent predictors of patients’ delay. Smear-negative TB (AOR, 1.88; 95 % CI, 1.32–2.68) and first visit to public health centers (AOR, 2.22; 95 % CI, 1.52–3.25) and health posts (AOR, 5.86; 95 % CI, 1.40–24.39) were found to be independent predictors of health system’s delay.

**Conclusions:**

The health system’s delay in this study was long and contributed more than 50 % of the total delay. Better TB diagnostic tools to complement sputum smear microscopy are needed to early diagnose PTB cases at peripheral health facilities. In addition, due emphasis should be given to increase public awareness about symptoms and consequences of TB disease.

**Electronic supplementary material:**

The online version of this article (doi:10.1186/s12879-016-1995-z) contains supplementary material, which is available to authorized users.

## Background

Tuberculosis (TB) is a major public health concern in the developing world. According to a recent World Health Organization (WHO) report, there were 9.6 million new TB cases and 1.5 million deaths from TB worldwide [1]. The 22 high TB burden countries collectively accounted for 80 % of all estimated incident cases.

Ethiopia is among the 22 high-TB burden countries in the world. The directly observed treatment short-course strategy (DOTS) has been adopted in the country since 1992 to control the TB epidemic [2]. The prevalence, incidence and mortality from all forms of TB in Ethiopia is currently estimated at 200/100,000 population, 207/100,000 population and 33 per 100,000 population, respectively [1]. These indicators show that the TB burden in Ethiopia is still enormous.

Early diagnosis and prompt initiation of treatment is an essential intervention to significantly reduce the TB burden. Diagnostic and treatment delays contribute to increased TB transmission and severity of illness [3]. Delay in diagnosis and treatment of TB has been studied in various parts of the world. A systematic review reported that the median time of delay from onset of cough until treatment initiation varied from 21 to 136 days [4]. A number of socio-demographic, economic, behavioral and clinical parameters were identified as factors associated with diagnostic delay [4].

Reports of earlier studies from different regions of Ethiopia showed median patients’ and health system’s delays ranging from 20 to 63 days and 6 to 42 days, respectively [5–15]. Various factors including rural residence [9, 11, 12], being smear-positive PTB case [9], being illiterate [9, 11, 14], being extra pulmonary TB (EPTB) case [12, 14], old age [6] and first visit to non-formal health provider [10–12, 14] were identified as predictors of long patients’ delay. On the other hand, first visit to health centers, health posts, clinics and private health facilities [10, 12], being EPTB case [8, 10] and far distance from health facilities [5] were associated with increased health system’s delay.

In Amhara Region where the current study was conducted, few studies assessed patients’ and health system’s delays [6, 12, 14, 15]. However, most of these studies [12, 14, 15] were done at health facilities found in the capital city of Amhara Region, Bahir Dar and did not consider peripheral health facilities. Only one study that was conducted in a comparable setting with the current study investigated diagnostic and treatment delay by including health institutions found in both rural and urban areas in Amhara Region [6]. However, the study subjects who were included in that study were only smear-positive cases, indicating the need for more studies.

The length and factors associated with patients’ and health system’s delays may greatly vary across different population, socio-economic conditions, culture and geographical settings. West Gojjam zone is one of the zones in the Amhara Region of Ethiopia where the highest number of TB cases were reported in 2013 [16]. Nonetheless, there is no study that addressed the magnitude and associated factors of patients’ and health system’s delays in this zone. Understanding the extent and factors associated with patients’ and health system’s delays in high TB burden settings helps to improve TB case detection, treatment and ultimately reduce the TB incidence. The aim of this study was to determine the length and analyze factors associated with patients’ and health system’s delays in the diagnosis and treatment of new PTB patients.

## Methods

### Setting

This study was conducted in West Gojjam Zone which is one of the ten zones of Amhara Region, Ethiopia. The total population is estimated at 2 382 497 [17]. More than 90 % of the population resides in rural areas, and agriculture is the main source of livelihood for the community. During the study period, one government hospital, 90 government health centers, 356 health posts and 76 private health institutions were rendering health services to the population. A health post is the lowest level health care and is staffed by two female health extension workers (HEWs). HEWs play an important role in identifying and referring TB suspects to the next level of health care i.e. health centers for TB diagnosis and initiation of treatment. Health posts are not equipped with TB diagnostic tools. Sputum smear microscopy is the only TB diagnostic tool for PTB patients attending majority of the health centers. Thus, clinically suspected smear-negative PTB cases are referred to hospitals and private health facilities for chest radiography and other tests for TB.

### Sampling technique

Random sampling method was used to select study sites. First, we obtained list of all public health facilities providing TB diagnostic and treatment services in West Gojjam Zone. Accordingly, 73 health centers and one hospital were providing TB diagnostic and treatment services during the study period. Of these, 29 health centers were randomly selected. We also added one hospital, which is the only available hospital in the study zone. This makes a total of 30 study sites. The private health facilities were not included in this study.

### Study design, population and data collection

This was a health facility based cross-sectional study conducted in 30 public health facilities in West Gojjam Zone from Oct 2013 to Oct 2014. All newly diagnosed PTB patients ≥ 15 years of age attending the selected sites were consecutively enrolled in the study. Participants were interviewed at the time of treatment initiation during the study period. PTB patients below 15 years of age, EPTB patients, and patients with MDR-TB and a previous history of TB were excluded from the study.

Socio-demographic and clinical data were collected using a pre-tested semi-structured questionnaire. The data among others included, age, sex, place of residence, symptoms at presentation, human immunodeficiency virus (HIV) sero-status, knowledge of TB, time period between onset of TB suggestive symptoms (i.e. cough, haemoptysis, weight loss, night sweats and fever) and first visit to formal-health provider, and time period between first visit to formal health provider and first start of treatment. Trained health officers and nurses at each study site collected the data. To assure quality of the data, frequent supervision was made by the principal investigator and other supervisors throughout the data collection period. Diagnosis of TB was based on the national TB diagnosis algorism [18]. Smear-positive PTB is diagnosed when a patient has at least two initial sputum smear examinations positive for acid-fast bacilli (AFB), or one initial smear examination positive for AFB and culture positive, or one initial smear examination positive for AFB and radiographic abnormalities consistent with active TB. Smear-negative PTB is diagnosed when a patient has symptoms suggestive of TB with at least three initial sputum smear examinations negative for AFB, radiographic abnormalities consistent with active PTB, no response to a course of broad spectrum antibiotics and a decision by a clinician to treat with a full course of anti-TB chemotherapy. Provider initiated HIV counseling and testing service is one of the routine clinical services given to TB patients.

Sample size was determined using the formula for estimating single population proportion. Therefore, by taking 48 % proportion of delay of more than 1 month from a previous study in Amhara Region [6], a margin error of 4 %, 95 % CI and 10 % non-response rate, the total sample size was estimated at 659. However, we included all of the 706 new PTB patients that attended the study sites during the study period. This accounted for 107 % of the minimum sample size required for the study.

### Operational definition of variables

A new case of TB is a patient who has never had treatment for TB or who had taken anti-TB drugs for less than 1 month.

Formal-health providers: modern health care facilities such as health posts, clinics, health centers and hospitals owned by the government or the private sector.

Non-formal health providers: include traditional health care providers and drug retail outlets.

Patients’ delay: the time period from onset of TB symptoms till first presentation to a formal- health provider.

Long patients’ delay: if the time period from the onset of TB symptoms to first presentation to a formal health provider exceeds more than 30 days.

Health system’s delay: the time period from first presentation to a formal health provider to first start of anti-TB treatment.

Long health system’s delay: if the time period from first presentation to a formal health provider to first start of anti-TB treatment exceeds more than 15 days.

Diagnostic delay: the time period from onset of TB symptoms to first diagnosis of TB.

Total delay: the time period from onset of TB symptoms to first start of anti-TB treatment.

### Statistical analysis

Data were entered, cleaned and analyzed using Statistical Package for the Social Sciences (SPSS) IBM Version 22 (SPSS Inc. Chicago, IL, USA). Descriptive statistics such as proportions, medians with IQR were computed. As the data were skewed, non-parametric tests (Mann–Whitney/Kruskal-Wallis) were employed to compare group differences in patients’ and health system’s delays. Mann–Whitney test was used to compare two groups and the Kruskal-Wallis test was used for comparing three or more groups. Based on experiences from previous studies, 30 days cut-off point was used to dichotomize patients’ delay into “delayed” and “non-delayed” patient groups [6, 12]. Likewise, 15 days cut-off point was applied to dichotomize health system’s delay into “delayed” and “non-delayed” patient groups [6, 12]. For each TB knowledge question, a score of one was given for the correct answer and a zero score was given for incorrect responses. Then, total knowledge score and median were calculated. Finally, those with a total score of below the median value were classified as having poor knowledge whereas, those equal or above the median value were considered as having good knowledge. To assess if patients felt perceived stigma due to TB, patients were asked a question related to TB related stigma. Those who answered yes were classified as having perceived stigma to TB whereas; those who answered no were classified as not having felt perceived stigma to TB.

In order to adjust the clustering effect and analyze the independent effects of each exposure variables on patients’ and health system’s delays, mixed-effect logistic regression model was used. Health facilities were held as random effect variable and other predictor variables were used as fixed effect.

To evaluate the applicability of the mixed-effects logistic regression model, the Intra-class Correlation Coefficient (ICC) was calculated in the empty model and it was indicated that 0.149 (14.9 %) of the total variance in health system delay was attributable to the differences across health facilities. The test of the preference of log likelihood Vs logistic regression was strongly significant (*P* < 0.001). Likewise, 0.043 (4 %) of the total variance in patients’ delay was attributable to the differences across health facilities. The test of the preference of log likelihood Vs logistic regression was significant (*P* = 0.01). Then, the full model was run by including health facilities as a random effect variable and other predictor variables as fixed effects and the (ICC) became 0.176 indicating that 17.6 % of the variation in health system’s delay was attributed to cluster level. The preference of log likelihood Vs logistic regression was still strongly significant (*P* < 0.001). Similarly, for the patients’ delay after the full model run the ICC was 0.062 indicating that 10.0 % of the variation in patients’ delay was attributed to cluster level. The preference of log likelihood Vs logistic regression was significant (*P* =0.05).

Univariate and multivariate analysis were performed using STATA V.14 (College station, TX USA). Variables found to be statistically significant in the bivariate analysis were included in multivariate model. In addition, variables that showed a *p* value of > 0.05 but were clinically or epidemiologically relevant for the analysis were included in the multivariate model. Crude odds ratio (COR) and AOR with 95 % CI were used to assess the degree of association between predictors and dependent variables. A *p*-value of less than 0.05 was considered statistically significant.

## Results

### Characteristics of the study subjects

A total of 706 new PTB patients were included in the study, and of these were 334 (47.3 %) sputum smear-positive and 372 (52.7 %) sputum smear-negative patients. The median age of the study participants was 30 years (IQR: 23–47 years), and 423 (59.9 %) were males. Nearly 62 % (435) of the study subjects reside in rural areas. More than 58 % (411) of patients had no formal education and 327 (46.3 %) were farmers by occupation. Majority, 95 % (671) of the study participants reported to have taken less than two hours to reach at the nearest health facility (Table 1).

**Table 1:** Socio-demographic and clinical characteristics of the study participants, West Gojjam Zone, Ethiopia

Variables	Number	Percent (%)
Age (years)		
15–24	197	27.9
25–44	291	41.2
≥ 45	218	30.9
Sex		
Male	423	59.9
Female	283	40.1
Place of residence		
Urban	271	38.4
Rural	435	61.6
Educational level		
Not literate	411	58.2
Literate	295	41.8
Marital status		
Married	386	54.7
Single	205	29.0
Divorced	73	10.3
Widowed	42	5.9
Occupation		
Civil servant	41	5.8
Housewife	39	5.5
Student	76	10.8
Farmer	327	46.3
Day laborer	82	11.6
Merchant	47	6.7
Others	94	13.3
Monthly family income (Birr) ^a^		
1–400	109	15.4
401–800	84	11.9
≥ 801	264	37.4
No regular income	249	35.3
HIV sero-status		
Positive	82	11.6
Negative	616	87.3
Not known	8	1.1
Time travelled to arrive at the nearest health facility		
≤ 2 h	671	95
> 2 h	35	5
Perceived to be stigmatized		
Yes	88	12.5
No	618	87.5
knowledge about TB		
Good	351	49.7
Poor	355	50.3
Types of PTB		
Smear-positive PTB	334	47.3
Smear-negative PTB	372	52.7

^a^ 1 USD = 22.00 Ethiopian Birr

*PTB* pulmonary tuberculosis

### TB symptoms and first health care seeking action

Majority, 97.9 % (619) of the patients had persistent cough. Other symptoms reported were: loss of appetite 633 (89.7 %), chest pain 578 (81.9 %), night sweats 569 (80.6 %), body weight loss 548 (77.6 %), fever 513 (72.7 %) and hemoptysis 167 (23.7 %).

Four hundred and thirty three (61.3 %) patients first sought health care from formal-health providers. Two hundred twenty three (31.6 %) patients initially sought health care from non-formal health providers. Among these, 112 (15.9 %) used holy water, 102 (14.4 %) visited drug retail outlets and 9 (1.3 %) sought treatment from traditional healers. The remaining 50 (7.1 %) patients first practiced self-treatment using various remedies at their homes. Among patients who first sought health care from formal-health providers, 236 (54.5 %) patients had no formal education. On the other hand, 175 (64.1 %) patients who first sought health care from non-formal health providers had no formal education (*P* < 0.01).

### Types of formal-health providers where all patients made their first consultation

Patients had reported the types of health facilities where they first sought health care. Accordingly, 342 (48.4 %), 71 (10 %), 13 (2 %) and 280 (39.6 %) initially visited public health centers, public hospitals, public health posts and private health facilities, respectively.

### Patients’ delay

**Fig. 1:**
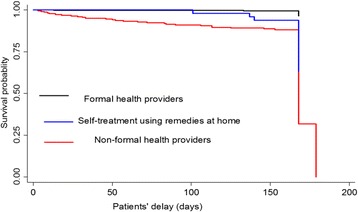
Kaplan-Meier survival estimate by first health care seeking action. *Note:* Patients’ delay is defined as the time period from onset of symptoms till first presentation to a formal-health provider. The graph describes patients’ delay by first visit to formal health provider, non-formal health provider and first practiced self-treatment with various remedies at their home

The median patients’ delay was 18 days (IQR: 8–34 days). The median patients’ delay for those patients who first visited non-formal health providers, formal-health providers, and those who first practiced self-treatment with various remedies at their home were 60 days, 14 days and 22 days, respectively (Kruskal-Wallis test; *p* < 0.001) (Table 2), (Fig. 1). Of the total study participants, 512 (72.5 %) patients visited formal-health providers within 30 days following the onset of symptoms. Of the study participants who first visited formal-health providers within 30 days after the onset of symptoms, 219 (42.8 %) had poor knowledge of TB compared to 136 (70.1 %) patients who first visited formal-health providers after 30 days of onset of symptoms and had poor knowledge of TB (*P* < 0.001).

**Table 2:** Patients’ and health system’s delays of new PTB patients by background characterstics, West Gojjam Zone, Ethiopia

Variables	N	Patients’ delay (days)			Health system’s delay (days)		
		Median	IQR	*P* value	Median	IQR	*P* value
Age (years)				< 0.001*			0.55
15–24	197	15.0	7–29		20.0	5–69	
25–44	291	17.0	7–31		21.0	5–81	
≥ 45	218	22.0	12–51		29.0	4–114	
Sex				0.33			0.95
Male	423	17.0	8–30		22.0	4–95	
Female	283	19.0	8–45		23.0	4–81	
Place of residence				0.001*			0.32
Urban	271	15.0	7–30		19	4–76	
Rural	435	20.0	9–45		25	4–100	
Educational level				< 0.001*			0.37
Not literate	411	21.0	12–55		26.0	4–109	
Literate	295	15.0	7–25		20.0	5–61	
Marital status				0.03*			0.58
Married	386	20.0	10–35		22.0	4–95	
Single	205	15.0	7–30		20.0	5–69	
Divorced	73	21.0	8–60		32.0	5–124	
Widowed	42	21.0	11–56		25.0	7–89	
Occupation				0.01*			0.71
Civil servant	41	7.0	5–15		24	6–49	
Housewife	39	21.0	7–60		7	3–27	
Student	76	15.0	7–30		19	4–58	
Farmer	327	20.0	9–40		26	4–113	
Day laborer	82	20.0	14–31		24	4–77	
Merchant	47	15.0	10–22		14	4–89	
Others	94	16.0	7–36		27	7–94	
Monthly family income (Birr) ^a^				0.35			0.22*
1–400	109	20.0	11–31		8.0	3–56	
401–800	84	19.0	11–62		22.0	5–78	
≥ 801	264	18.0	7–31		27.0	4–113	
No regular income	249	16.0	7–36		24.0	4–73	
HIV sero-status				0.68			0.31
Positive	82	20.0	11–45		22.0	5–86	
Negative	616	18.0	8–33		22.0	4–88	
Not known	8	16.0	7–29		74.0	8–320	
Time travelled to the nearest health facility				0.08			0.96
≤ 2 h	671	17.0	8–31		23	4–88	
> 2 h	35	23.0	14–70		15	4–106	
Perceived to be stigmatized				0.47			0.70
Yes	88	18.0	8–31		19.0	4–87	
No	618	19.5	10–39		23.0	4–88	
knowledge about TB				< 0.001*			0.14
Good	351	15.0	7–25		18.0	5–61	
Poor	355	23.0	14–60		28.0	4–113	
Types of PTB				0.23			< 0.001*
Smear-positive PTB	334	16.0	8–31		15	3–76	
Smear-negative PTB	372	19.0	7–39		27	7–95	
First health care seeking action				< 0.001*			0.12
Visited formal health providers	433	14	7–21		24	5–93	
Visited non -formal health providers	223	60	24–116		15	4–86	
Self-treatment using various remedies at home	50	21.5	15–60		27	4–85	
Type of formal health provider first visited				0.81			0.02*
Private health facilities	280	20	8–31		15	4–70	
Public health centers	342	15	7–37		31	5–97	
Public hospitals	71	18	10–41		15	4–86	
Health posts	13	15	8–30		29	10–203	

*Note:* using non-parametric Kruskal-Wallis test to compare three or more groups and Mann–Whitney test to compare two groups * statistically significant. *IQR* inter quartile range, *PTB* pulmonary tuberculosis, *HIV* human immunodeficiency virus

In the multivariate analysis, patients who first visited non-formal health providers and those who first practiced self-treatment with various remedies at their home were more likely to experience longer patients’ delay compared to patients who first visited formal-health providers (AOR, 47.56; 95 % CI, 26.31–85.99) and (AOR, 10.11; 95 % CI, 4.53–22.56), respectively. Being in the age group ≥ 45 years, and those patients who had poor knowledge of TB were more likely to experience longer patients’ delay compared to the younger age group and patients with good knowledge of TB (AOR, 2.99; 95 % CI, 1.14–7.81) and (AOR, 2.33; 95 % CI, 1.34–4.05), respectively (Table 3).

**Table 3:** Associations of socio demographic and clinical factors with patients’ delay among PTB patients, West Gojjam Zone, Ethiopia

Variables	N	Delayed n (%)^¶^	Crude OR (95 % CI)	*P* value	Adjusted OR (95 % CI)	*P* value
Sex						
Male	423	103 (24.3)	1.00		1.00	
Female	283	91 (32.2)	1.54 (1.09–2.19)	0.01*	1.25 (0.72–2.15)	0.43
Age						
15–24	197	39 (19.8)	1.00		1.00	
25–44	291	77 (26.5)	1.45 (0.93–2.28)	0.09	2.27 (0.96–5.38)	0.06
≥ 45	218	78 (35.8)	2.30 (1.45–3.64)	< 0.001*	2.99 (1.14–7.81)	0.03*
Education						
Literate	295	55 (18.6)	1.00		1.00	
Not literate	411	139 (33.8)	2. 22 (1.54–3.21)	< 0.001*	0.87 (0.44–1.72)	0.69
Occupation						
Civil servant	41	3 (7.3)	1.00		1.00	
Housewife	39	14 (35.9)	7.16 (1.82–28.08)	0.01*	1.04 (0.13–8.29)	0.97
Student	76	18 (23.7)	4.33 (1.16–16.06)	0.03*	1.09 (0.15–7.75)	0.93
Farmer	327	102 (31.2)	5.82 (1.73–19.61)	0.01*	0.87 (0.17–4.32)	0.87
Day laborer	82	21 (25.6)	4.46 (1.22–16.24)	0.02*	0.61 (0.98–3.78)	0.59
Merchant	47	9 (19.1)	2.97 (0.72–12.11)	0.13	0.58 (0.90–3.74)	0.57
Others	94	27 (28.7)	5.46 (1.52–19.57)	0.01*	0.65 (0.99–4.33)	0.66
Marital status						
Married	386	109 (28.2)	1.00		1.00	
Single	205	44 (21.5)	0.72 (0.47–1.08)	0.12	1.02 (0.44–2.33)	0.96
Divorced	73	24 (32.9)	1.27 (0.73–2.21)	0.39	1.23 (0.52–2.88)	0.64
Widowed	42	17 (40.5)	1.92 (0.97–3.81)	0.06	2.01 (0.68–5.90)	0.20
Place of residence						
Urban	271	59 (21.8)	1.00		1.00	
Rural	435	135 (31.0)	1.61 (1.11–2.33)	0.01*	1.60 (0.87–2.94)	0.13
Monthly family income (Birr) ^a^						
1–400	109	30 (27.5)	0.96 (0.57–1.63)	0.91	0.82 (0.27–2.45)	0.73
401–800	84	28 (33.3)	1.22 (0.71–2.13)	0.46	0.76 (0.23–2.56)	0.66
≥ 801	264	67 (25.4)	0.82 (0.54–1.24)	0.36	0.61 (0.19–1.89)	0.39
No regular income	249	69 (27.7)	1.00		1.00	
HIV sero-status						
Negative	616	169 (27.4)	1.00		1.00	
Positive	82	24 (29.3)	1.06 (0.63–1.79)	0.82	0.76 (0.34–1.71)	0.52
knowledge about TB						
Good	351	58 (16.5)	1.00		1.00	
Poor	355	136 (38.3)	3.39 (2.33–4.95)	< 0.001*	2.33 (1.34–4.05)	0.01*
Perceived to be stigmatized						
No	618	167 (27.0)	1.00		1.00	
Yes	88	27 (30.7)	1.18 (0.71–1.98)	0.51	1.37 (0.66–2.84)	0.39
Time travelled to arrive at the nearest health facility						
≤ 2 h	671	181 (27.0)	1.00		1.00	
> 2 h	35	13 (37.1)	1.64 (0.78–3.43)	0.18	0.65 (0.23–1.79)	0.40
First health care seeking action						
Visited formal health provider	433	24 (5.5)	1.00		1.00	
Visited non-formal health provider	223	152 (68.2)	39.73 (23.39–67.48)	< 0.001*	47.56 (26.31–85.99)	< 0.001*
Self-treatment using various remedies at their homes	50	18 (36.0)	9.97 (4.79–20.76)	< 0.001*	10.11 (4.53–22.56)	< 0.001*
Types of PTB						
Smear -positive PTB	334	84 (25.1)	1.00		1.00	
Smear -negative PTB	372	110 (29.6)	1.30 (0.93–1.84)	0.13	1.32 (0.81–2.16)	0.26

Estimated for random effect =0.384 (0.197–0 .746)

^¶^ Delayed more than 30 days, ^a^ 1 USD = 22.00 Ethiopian Birr *statistically significant association, *PTB* pulmonary tuberculosis, *N* number, *OR* odds ratio, *CI* confidence interval

### Health system’s delay

**Fig. 2:**
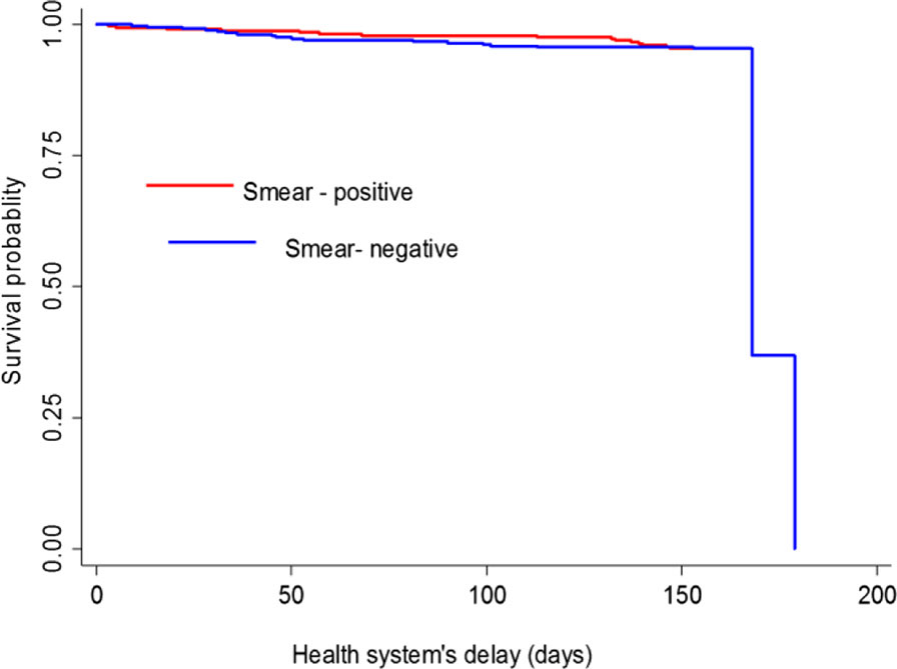
Kaplan-Meier survival estimate by TB type. *Note:* Health system’s delay defined as the time period from first presentation to a formal health provider to first start of anti-TB treatment. The graph describes health system’s delay by type of TB

The median health system’s delay was 22 days (IQR: 4–88 days). The health system’s delay for 394 (55.8 %) study participants was more than 15 days. In bivariate analysis, smear-negative patients had longer health system’s delay than smear-positive patients: 27 days versus 15 days, respectively (Mann–Whitney test, *P* < 0.001) (Table 2), (Fig. 2).

In multivariate analysis, smear-negative patients were more likely to experience longer health system’s delay than smear-positive patients (AOR, 1.88; 95 % CI, 1.32–2.68). Patients who first visited public health centers and health posts were more likely to experience longer health system’s delay compared to patients who first visited private health institutions (AOR, 2.22; 95 % CI, 1.52–3.25) (AOR, 5.86; 95 % CI, 1.40–24.39), respectively. Patients who were housewife by occupation were less likely to experience longer health system’s delay compared to civil servants (AOR, 0.10; 95 % CI, 0.02–0.40) (Table 4).

**Table 4:** Associations of sociodemographic and clinical factors with health system’s delay among PTB patients, West Gojjam Zone, Ethiopia

Variables	N	Delayed ^a^ n (%)	Crude OR (95 % CI)	*P* value	Adjusted OR (95 % CI)	*P* value
Sex						
Male	423	236 (55.8)	1.00		1.00	
Female	283	158 (55.8)	1.00 (0.72–1.39)	0.99	1.11 (0.76–1.61)	0.57
Age						
15–24	197	110 (55.8)	1.00		1.00	
25–44	291	162 (55.7)	0.96 (0.64–1.42)	0.85	0.85 (0.51–1.44)	0.56
≥ 45	218	122 (56.0)	1.03 (0.67–1.58)	0.86	0.85 (0.47–1.53)	0.58
Education						
Literate	295	165 (55.9)	1.00		1.00	
Not literate	411	229 (55.7)	1.12 (0.81–1.57)	0.47	1.25 (0.78–1.99)	0.35
Occupation						
Civil servant	41	25 (61.0)	1.00		1.00	
Housewife	39	11 (28.2)	0.21 (0.07–0.58)	0.01*	0.10 (0.02–0.40)	0.01*
Student	76	43 (56.6)	0.77 (0.33–1.82)	0.56	0.39 (0.11–1.35)	0.14
Farmer	327	187 (57.2)	0.89 (0.43–1.85)	0.76	0.85 (0.35–2.04)	0.73
Day laborer	82	47 (57.3)	0.78 (0.34–1.81)	0.57	0.53 (0.17–1.64)	0.27
Merchant	47	22 (46.8)	0.57 (0.22–1.46)	0.24	0.91 (0.32–2.59)	0.86
Other	94	59 (62.8)	1.04 (0.45–2.38)	0.92	0.62 (0.19–2.01)	0.43
Place of residence						
Urban	271	144 (53.1)	1.00		1.00	
Rural	435	250 (57.5)	1.38 (0.97–1.96)	0.07	1.24 (0.80–1.93)	0.33
Monthly family income (Birr) ^b^						
1–400	109	50 (45.9)	0.69 (0.42–1.14)	0.15	0.46 (0.18–1.03)	0.06
401–800	84	43 (51.2)	0.85 (0.49–1.47)	0.57	0.47 (0.19–1.17)	0.11
≥ 801	264	157 (59.5)	1.15 (0.78–1.71)	0.46	0.60 (0.25–1.42)	0.25
No regular income	249	144 (57.8)	1.00		1.00	
HIV sero- status						
Positive	82	47 (57.3)	1.05 (0.63–1.74)	0.86	1.33 (0.72–2.46)	0.35
Negative	616	342 (55.5)	1.00		1.00	
Time travelled to arrive at the nearest health facility						
≤ 2 h	671	377 (56.2)	1.00		1.00	
> 2 h	35	17 (48.6)	0.85 (0.41–1.78)	0.67	0.68 (0.31–1.51)	0.35
Formal health providers ^c^						
Private health facilities	280	137 (48.9)	1.00		1.00	
Public health centers	342	213 (62.3)	2.00 (1.40–2.85)	< 0.001*	2.22 (1.52–3.25)	< 0.001*
Public hospitals	71	34 (47.9)	0.86 (0.48–1.55)	0.63	0.79 (0.43–1.48)	0.47
Health posts	13	10 (76.9)	4.75 (1.17–19.27)	0.03*	5.86 (1.40–24.39)	0.02*
Forms of PTB						
Smear-positive PTB	334	166 (49.7)	1.00		1.00	
Smear-negative PTB	372	228 (61.3)	1.64 (1.18–2.27)	0.01*	1.88 (1.32–2.68)	< 0.001*

Estimated for random effect = 0.702 (0 .337, 0.965)

^a^ Delayed more than 15 days, *OR* odds ratio, *CI* confidence interval

^b^ 1 USD = 22.00 Ethiopian Birr *statistically significant association, *PTB* pulmonary tuberculosis

^c^ Types of formal-health providers where all patients made their first consultation

### Total delay

The median total delay was 60 days. Health system’s delay contributed the largest portion of the total delay.

## Discussion

In this study, the health system’s delay contributed more than 50 % of the total delay. Interventions that may reduce health system’s delay have been put in place in the study area. TB screening activities have been integrated with the general health service in health care facilities. According to the national TB control guideline [18], patients presenting with cough of more than 2 weeks are subjected to smear microscopy examination for AFB. This strategy helps to minimize missed opportunities for early diagnosis and treatment initiation for TB patients [19]. In addition, the DOTS strategy has been decentralized, making TB diagnostic and treatment services more accessible to the community. Increased health services accessibility reduces both patients’ [20, 21] and health system’s delays [5]. Despite the integration of TB screening activities in the general health care and increased access in TB diagnostic and treatment services however, it is surprising that the median health system’s delay observed in the current study is still similar with findings of former studies conducted in Amhara Region of Ethiopia [6, 12]. In a recent qualitative study [22], a number of challenges that may have implications for increased health system’s delay were reported from the study area. Among others, frequent interruptions of laboratory reagents and anti-TB drugs supplies, lack of diagnostic tools for smear-negative TB and lack of laboratory personnel in some health centers were identified as challenges of the TB control program [22]. Interventions directed at solving these challenges are essential to reduce the long health system’s delay observed in the study area.

Studies in other regions of Ethiopia (Addis Ababa [5], Southern Region [13]) showed shorter median health system’s delay compared to the current study result. This may be due to the differences in study settings. The studies in Addis Ababa and Southern Region of Ethiopia were conducted in urban setting where better diagnostic facilities and experienced health care providers are available. The median health system’s delay in our study is also higher than findings of similar studies done in other African countries such as Angola [23] and Zimbabwe [24].

The median patients’ delay observed in this study is 18 days. This is shorter than the findings of former studies conducted in comparable settings in Amhara Region (median patients’ delay 30 days) [6, 7]. It is also shorter compared to findings of similar studies from other regions of Ethiopia: Addis Ababa (median patients’ delay 60 days [5], Tigray (median patients’ delay 30 days) [9] and Southern Region (median patients’ delay 30 days) [13]. The median patients’ delay in this study is comparable with findings of former studies from other countries such as Malawi (median patients’ delay 14 days) [21] and India (median patients’ delay 15 days) [25]. The discrepancies in the study findings might be related to differences in study settings, sample sizes used, socio-cultural and socio-demographic factors, definitions and cut-off points used to measure the delay periods and study time periods. The relatively shorter median patients’ delay observed in our study may be related to improved access to TB diagnostic and treatment services as a consequence of DOTS decentralization [26]. It may also be linked to the increasing involvement of HEWs in identifying and referring TB suspects to the nearest health facilities where AFB smear microscopy test is available.

More than half of the study participants (72.5 %) sought health care from formal-health providers within 30 days following the onset of TB symptoms. In a previous study conducted in a comparable setting in Amhara Region, 52 % of the study participants visited formal-health providers within 30 days [6]. This may indicate patients increased preference to formal-health providers for health seeking.

The health system’s delay for 394 (55.8 %) study participants exceeded 15 days. Patients who first consulted public health centers experienced longer health system’s delay compared to those who first visited private health facilities. This is in line with other study findings in Ethiopia [10] and Malawi [21]. On the other hand, longer health system’s delay among TB patients who first visited private medical providers than those who consulted public health center was reported in a study conducted in Amhara Region, Ethiopia [6]. This difference may be related to the increasing involvement of private health facilities in TB care in the last 6 years in the study region.

The long health system’s delay observed among patients who first visited public health centers in the current study may be due to lack of better TB diagnostic tools at health centers. Smear microscopy is the only TB diagnostic tool used in majority of health centers of the study area during the study period. Smear microscopy has very low sensitivity [27] and many patients can get false negative results. The long health system’s delay may also be related to less experienced health providers particularly working at peripheral health centers [22]. Thus TB patients may require to make repeated visits before they are diagnosed and started on treatment. A previous systematic review indicated that the problem of delay in diagnosis and treatment of TB is a vicious cycle of repeated visits at the same health care level [4].

First vist to health posts was also identified as a factor for longer health system’s delay. This is similar with findings of previous studies done in Ethiopia [6, 10]. The finding can be explained by the fact that health posts are not equipped with diagnostic facilities for TB. Health posts are run by HEWs whose roles are limited to identifying and referring TB suspects to the nearest health centers where smear microscopy test for TB is available.

Being a smear-negative TB case is another factor associated with health system’s delay. Similar findings were observed in other countries [21, 28]. Diagnosis of PTB in the study zone is based on the national TB diagnostic and treatment guideline [18]. According to the guideline, PTB suspects with smear-negative TB are first treated with non-specific broad-spectrum antibiotics for 7-10 days duration. Subsequently, patients will be re-examined within 2-4 weeks before they are started on anti-TB drugs. This indicates that the diagnostic process for smear-negative TB patients may take between 15 and 30 days before anti-TB treatment is initiated. Delay in diagnosis of smear-negative TB has significant contribution to TB transmission in the community. Previous studies from Canada and California demonstrated that the contribution of patients with smear-negative, culture-positive TB to TB transmission were 17 % and 22 %, respectively [29, 30]. Based on the national TB prevalence survey in Ethiopia, 57 % of smear-negative cases were culture positive [31]. Another study in Ethiopia documented that 47 % smear-negative cases were culture positive [32]. This may suggest that availing better diagnostic tools for health institutions, particularly at health centers where majority of the TB patients first seek care expedites timely diagnosis and treatment of PTB cases.

The level of knowledge about TB was found to be significantly associated with patients’ delay. Patients who had poor knowledge of TB were more likely to have longer patients’ delay than those who had good knowledge of TB. This is consistent with findings of previous studies [8, 9, 21, 33, 34] and may suggest that lack of awareness may lead to patients’ reluctance in seeking appropriate health care.

A patient with 2 weeks duration of persistent cough is considered a suspected case of TB [35]. In this study, nearly 28 % of the study participants made their first consultation with formal-health providers after staying more than 30 days following onset of symptoms. This finding suggests that concerted effort is required to increase public awareness about the importance of early health care seeking from formal-health providers.

Patients who first visited non-formal health providers had longer patients’ delay compared to those who first consulted formal-health providers. This is similar with findings of previous studies in Ethiopia [10–12, 14] and other African countries [24, 33]. More than 30 % of patients initially sought traditional form of health care before visiting formal health providers. This finding is in line with previous studies [6, 13]. Our finding may indicate that a substantial proportion of TB patients are still first using informal treatment for TB symptom management. This may have implications on severity of TB, poor treatment outcome and transmission of TB in the community. Training non-formal health providers about TB suspect identification and early referral may contribute to reduce patients’ delay.

Patients, who first practiced self-treatment with various remedies at their home, were more likely to experience longer patients’ delay than those who first visited formal-health provides. This is similar with reports of other studies [6, 10], which might be related to poor knowledge of TB. Patients in the age group ≥ 45 years experienced long patients’ delay compared to the younger age group which is consistent with other studies [6, 24]. Older patients are dependent on others help and may not early seek health care [12]. In addition, patients in the older age group usually present with non-specific symptoms making it difficult to diagnose TB [12, 36]. The reason for the observed lower health system’s delay among housewives compared to civil servants is not clear and warrants for further study.

The study has potential limitations. Firstly, it did not include extra pulmonary and retreatment TB cases. Secondly, the study was carried out only in government health facilities. Private health facilities were not included. Therefore, the results cannot be generalized to all TB patients attending public and private health facilities in the study area. Patients may not exactly remember the onset of their symptoms and is subject to recall bias. However, efforts were made to reduce recall bias by using local calendars listing major religious and national days to define the perceived date of onset TB symptoms.

## Conclusions

A substantial proportion of PTB patients in this study were delayed before diagnosis and initiation of treatment. The health system’s delay was long and contributed more than 50 % of the total delay. Smear-negative TB, first visit to public health centers and health posts were predictors for long health system’s delay. Poor knowledge of TB, patients’ age ≥45 years of age, self-treatment and first health seeking from informal-health providers were factors associated with longer patients’ delay. The study highlights the need to enhance TB diagnostic capacity of health facilities particularly public health centers. Better TB diagnostic tool to complement (AFB) sputum smear microscopy is needed to early diagnose PTB cases at peripheral health facilities. In addition, due emphasis should be given to increase public awareness about the symptoms and consequences of TB disease.

## Additional files

Additional file 1: Data set used for the article. The data set consists of 706 cases included in the study and the variables used in this article. (XLS 418 kb)
Additional file 2: A questionner for the study. A semi-structured questionnaire has been developed for the study. As described in the methodology section the questionnaire was pre-tested before commencement of data collection. (DOCX 48 kb)

## Abbreviations

AFB: Acid-fast bacilli
AOR: Adjusted odds ratio
CI: Confidence interval
COR: Crude odds ratio
DOTS: Directly observed treatment short course
EPTB: Extra pulmonary tuberculosis
HEWs: Health extension workers
HIV: Human immunodeficiency virus
IQR: Interquartile range
MDR-TB: Multidrug resistant tuberculosis
OR: Odds ratio
PTB: Pulmonary tuberculosis
SPSS: Statistical package for social sciences
TB: Tuberculosis
USD: United State Dollar
WHO: World Health Organization
