# Supplementary material for: Patients’ and health system’s delays in the diagnosis and treatment of new pulmonary tuberculosis patients in West Gojjam Zone, Northwest Ethiopia: a cross-sectional study
Source: BMC Infect Dis. 2016 Nov 11;16:673. doi: 10.1186/s12879-016-1995-z (PMC5106835; doi:10.1186/s12879-016-1995-z)
Supplement: Additional file 2: — A questionner for the study. A semi-structured questionnaire has been developed for the study. As described in the methodology section the questionnaire was pre-tested before commencement of data collection. (DOCX 48 kb) [file 12879_2016_1995_MOESM2_ESM.docx]

Appendix

Patients’ and health system’s delay among new pulmonary TB patients: Patient interview questionnaire.

Questionnaire no. _____________ Date________________________________

Name of Health Institution: ____________________________________________

Interviewer _________________________

1. Socio demographic information

1.1 Patient name ________________________________

1.2 Address: Region_________ Zone__________ Woreda _________ Kebele_________

1.3 Place of residence: Urban_______ Rural________

1.4 Age in years _______

1.5 Sex: Male_____ Female_____

1.6 Marital status:

Single___________ Divorced____________

Married___________ Widowed___________

1.6 Educational status

| Educational level | Mark ‘‘X’’ in front of patient response |
| --- | --- |
| Unable to read and write |  |
| Grade 1-4 |  |
| Grade 5-8 |  |
| Grade 9-10 |  |
| Grade 11-12 |  |
| Above grade 12 |  |
| Other (Specify) |  |

1.7 Average monthly income of the family in Eth.Birr

1-200 Birr________201-400 Birr _________401-600 Birr _______601-800 Birr________

801 and above ________ No defined monthly income __________

1.8 Occupation

| Occupation | Mark ‘‘X’’ in front of patient response |
| --- | --- |
| Civil servant |  |
| Housewife |  |
| Student |  |
| Farmer |  |
| Day laborer |  |
| Merchant |  |
| others (please specify) |  |

2. Suggestive TB symptoms

2.1 Do you have cough?

Yes________ No_________

2.2 Do you have sputum?

Yes_________ No________

2.3 If the patient’s answer is yes for question number 2.2 please ask if there is blood in sputum.

Yes_________ No_______

2.4 Do you have shortness of breathing?

Yes______ No______

2.5 Do you have chest pain?

Yes_________ No ______

2.6 Do you have fever?

Yes ______No ______

2.7 Do you have body weight loss?

Yes__________ No_________

2.8 Do you have night sweats?

Yes ________ No________

2.9 Do you have loss of appetite?

Yes_________ No_______

2.10 When did your current symptoms start? ______/_____/_____write (the date/month/year)

3. Health seeking pattern of the patient

3.1 When you first started to you have symptoms of the current illness, what did you do first?

| No | What did you do first | Mark ‘‘X’’ in front of patient response |
| --- | --- | --- |
| 1 | Self-treatment using homemade remedies |  |
| 2 | Visited drug retail outlets |  |
| 3 | Visited traditional healer |  |
| 4 | Used holy water |  |
| 5 | Visited modern health facility (health post, public health center, public hospital, private hospital, private clinic) |  |
| 6 | Other (please specify) |  |

3.2 Formal healthcare provider the patient first visited after onset of current symptoms?

| No | Health facilities | Mark ‘‘X’’ in front of patient response |
| --- | --- | --- |
| 1 | Health post |  |
| 2 | Public health center |  |
| 3 | Public hospital |  |
| 4 | Private hospital |  |
| 5 | Private clinic |  |

4. Walking distance from patient residence to the nearest public health facility

1 hour or less_____________ 1-2 hours_________________

3-4 hours ___________________More than 4 hours_________________

5. Time of diagnosis and treatment start:

5.1 When did you first visit to a formal health care provider for the current illness _____/____/_______ (write date/month/year)

5.2 When did first diagnosis for TB at health facility ______/______/_______ (write date/month/year)

5.3 When did first initiation of anti-TB drugs at the current health facility_____/______/______ (Write date/month/year)

5.4 Time interval from the start of suggestive TB symptoms till first visited to a formal health care provider?

_______days

5.5 Time interval from first visit to a formal health care provider till first diagnosis of TB?

_______days

5.6 Time interval from first diagnosis of TB till first start of anti-TB drugs?

_______days

5.7 Time interval from first start of suggestive TB symptoms till first start of anti-TB drugs?

_______days

6. Knowledge about Tuberculosis:

6.1 Have you ever heard about TB?

Yes______ No ______

6.2 Do you know the symptoms of TB?

Yes______ I don't know______

6.3 If yes please tell us what you know? (Mark ‘‘X’’ those mentioned by the patient)

Cough_____

Bloody sputum_______

Night sweat __________

Fever __________

Body weight loss_________

6.4 What do you think is the cause of TB? ___________________________________

6.5 Do you think TB can transmit from one person to another?

Yes________

No_______

I don't know___________

6.6 Do you think TB is curable?

Yes ____________

No _____________

I don’t know _______

7. Do you think that you would be stigmatized because of TB?

Yes__________

No___________

I don’t know__________

|  |
| --- |

8. TB disease classification: PTB+ve ________ PTB-ve________

9. Date/month/year when the patient started TB treatment _____/_____/______

10. HIV sero-status:

10.1 Positive____________

10.2 Negative____________

10.3 Unknown____________

*Thank you for your time and valuable information!*
